# Wymysorys revitalisation theatre: identity and community (re)building

**DOI:** 10.1080/23311983.2025.2489840

**Published:** 2025-05-02

**Authors:** Justyna Majerska-Sznajder, Joanna Maryniak, Justyna Olko

**Affiliations:** a Museum of Vilamovian Culture and Center for Research and Practice in Cultural Continuity, Faculty of “Artes Liberales”, University of Warsaw, Wilamowice, Poland; b Center for Research and Practice in Cultural Continuity, Faculty of “Artes Liberales”, University of Warsaw, Łódź, Poland; c Center for Research and Practice in Cultural Continuity, Faculty of “Artes Liberales”, University of Warsaw, Warszawa, Poland

**Keywords:** Vilamovian, theatre, language revitalisation, endangered languages, The Little Prince, The Hobbit, Community theatre, drama education & drama therapy, world theatre, bilingualism & multilingualism, language & power, interdisciplinary language studies, translation & interpretation

## Abstract

The small town of Wilamowice in southwestern Poland is home to the ethnic group of Vilamovians who speak their own endangered language: Wymysiöeryś. Amateur theatre created by the troupe *Ufa fisa* has been one of the most important and outwardly visible tools used in the process of revitalising this language. Its members, drawn from the town youth, perform various pieces, including adaptations of world literature such as *The Little Prince* or *The Hobbit*, their own creations based on local knowledge and historical memory (*Ymertihła* and *Ojeruma*) as well as an adaptation of the most important Vilamovian literary monument, Florian Biesik’s *Uf jer wełt* (the Vilamovian version of Dante’s *Divine Comedy*). This paper situates the Vilamovian revitalisation theatre in the broader context of similar endeavours in other endangered languages all around the globe. It then discusses in detail the creation of each play putting a special emphasis on the meaning of the piece for the community members and for the outside public. The impact of the long-term theatrical activity is later examined through the lens of philological approach as well as qualitative and quantitative research conducted in the community, showcasing the qualitative differences among the three generations of Vilamovians and the quantitative correlations between various kinds of engagement with Vilamovian culture. We argue that this performative art has been a vital instrument in language reclamation, in challenging the oppressive nation-state ideologies and the co-creation of a modern Vilamovian identity along with the process of (re)building of the community.

## Positionality and goals

Our own positionality is important in the context of this paper. Justyna Majerska-Sznajder, of the (unrecognised) Vilamovian ethnic minority, is a community-based language activist, emic researcher and educator; Joanna Maryniak and Justyna Olko are academics working within a multidisciplinary, self-reflective paradigm and, at the same time, are informed and engaged allies of various minority/Indigenous communities with whom they have collaborated over the long term in language revitalisation, education and minority (language) rights advocacy. In particular, we have been actively engaged in the revitalisation of Wymysiöeryś as allies of local activists. The reflections, results and data we bring together in this paper are an important part of our methodology and research transparency, which builds on input from several complementary disciplinary areas, all referring to and/or relevant for language revitalisation and reclamation: sociolinguistics, linguistic anthropology, art, history, language education and language policy.

In terms of theoretical approach, we wish to highlight the importance of locality and grassroots perspectives for language reclamation and identity building, going beyond the general Global South – Global North discourse, as there is no ontological or epistemological universality within the so-called Global North with regard to its own internal diversity and epistemological violence toward ethnic minorities within its territories. But nation state paradigms and different forms of epistemological violence still persist, including in approaches to minority language education and support. This is also relevant for how artistic creation and expression are perceived in relationship to social action, as well as for knowledge production and transmission. Southern theory, understood as ‘non-Eurocentric ways of knowing’, has been fundamental for decolonial turns in linguistics and other disciplines through its disassociation from western ways of creating knowledge, but it is not linked to any specific geographic region (Deumert & Makoni, 2023, p. 5). Therefore, our paper is about a place, Wilamowice, that, together with its people, has been part of a process of knowledge (re)creation and (re)transmission, but also an object of violence, carrying deep cultural wounds (see Kearney 2020). A deeper understanding of entanglements with local history is also an essential component of our approach. We find this particularly relevant for a society that has undergone forced homogenisation through imposed monolingual practices and that continues to be very much affected by its experience of historical trauma and ethnic discrimination. While speakers of minority languages have been ‘often shamed into silence’ (Coulmas, 2018, p. 69), there is a growing awareness that in order to develop and protect positive place-based identity, it is not enough to protect the language; positive identification, crucial for the wellbeing of minoritised communities, should also be (re)built and strengthened using cultural, symbolic, artistic, social and intellectual resources. Historical harm must be addressed and communities must pursue their own decolonial historical narratives, as well as their epistemological autonomy. In Wilamowice this harm has been addressed through collective language reclamation efforts and revitalisation theatre. This has had an enormous internal and external impact and resonance because community-driven expression, with their own discourse and collective practice focused on relational identities and ethnic differences, allows ‘the voice of the other, after being introduced into the postcolonial discourse, to become audible enough to achieve a readiness to dialogue with the voice of the center’ (Duć-Fajfer, 2006, p. 447). This approach on the part of the Vilamovian community has addressed the complex needs and challenges – epistemological, cultural, linguistic, educational, social, health-related and even political – faced by the collectivity and its painful experiences with external and internal forms of colonialism.

The case of Wilamowice and its linguistic-cultural revitalisation shows how history can be a tool of both oppression and healing. The transgenerational trauma present in this community is still perpetuated today by the ‘professional history writing’ of external researchers, who are self-confident enough even to claim, in the official and commune-sponsored monograph on the town, that ‘by signing (or consenting to sign) the Deutsche Volksliste they recognised themselves as Germans’ (Fic, 2018, p. 334). For the members of the community, such narratives trigger their historical trauma and they recognise it as epistemological violence committed by external researchers, who ignore local perspectives and the inhabitants’ complex, relational sense of identity (Król, 2018a, p. 417). A response to these imposed historical narratives has come directly from younger community members, who are actively learning and speaking the Wymysiöeryś language, and it has become part of language reclamation efforts. One of the key moments in this community-driven reclamation and identity (re)building was the initiative of new teenage speakers of Wymysiöeryś and members of the local amateur theatre troupe *Ufa fisa* to write and stage a play, *Ymertihła*. Addressing and confronting painful memories and dominant narratives coming from the outside, they challenged the imposed vision, highlighting their epistemological autonomy and language reclamation efforts. The play, performed both in Wilamowice and in the Polish Theatre in Warsaw, has also been an act of self-empowerment and identity building for the emerging Vilamovian community of practice. This approach finds close parallels and affinity to other initiatives involving the linking of theatrical art to language reclamation in Indigenous communities. Thus, in Native American and Hawaiian language reclamation efforts, the theatre became a tool of learning and teaching, but also an efficient means for the transmission and protection of lifeways, cultural and spiritual values and, above all, a space for healing (e.g. Baker, 2018; Driskill, 2003).

This paper is organised in the following way. After a brief introduction to Wilamowice, its recent history and the challenges associated with linguistic and cultural survival, we discuss the previous writings on the history of Vilamovian theatre and go on to describe in detail all the plays staged by the local theatre group *Ufa fisa*, along with the circumstances of their creation, their contents and their significance for the local community, with a special focus on assessing their impact on language reclamation, as well as on community and identity (re)building. In the next section we present the reactions of the community and feedback from community members of different age groups with regard to the local language revitalisation theatre, divided into qualitative and quantitative feedback. In the final section we provide a concluding discussion and interpretations.

## Wilamowice: its people and language

Wilamowice is a town located in southern Poland, in the Silesian Province, between Bielsko-Biała and Oświęcim. It has over 3,000 inhabitants and covers an area of approximately 10 square kilometres. The town is inhabited by the descendants of thirteenth-century Western European settlers – Vilamovians. They use the Wymysiöeryś language belonging to the western group of Germanic languages (Król, 2016a; Wicherkiewicz, 2003; Wicherkiewicz et al., 2018). Despite their cultural differences concerning the Slavic environment, Vilamovians are not formally recognised by the Polish government. Similarly, despite many years of efforts by local activists, inhabitants of the town, researchers and external experts, the Wymysiöeryś language has not been officially recognised as a language, so it does not currently have any legal protection, which is critically needed today (Król, 2019). The intergenerational transmission of the language was brutally interrupted by the ban on its use, issued in 1945, and the resulting persecutions. They started very harshly and included incarceration in post-war labour camps, deportations into Soviet territory beyond the Ural Mountains, evictions from households, expropriation of farms and many other forms of oppression, especially physical violence toward women. This violent period particularly affected the youngest children, whose first language was Wymysiöeryś and who had to hide and suppress speaking it. Over time, the repressions have diminished, but until the 1990s there was widespread discrimination and bullying directed at the Vilamovians. The official justification for this post-war violence was that some of the residents of the town had signed the Volksliste during the Nazi occupation, which was, in theory, voluntary for Poles, but mandatory for the alleged ethnic Germans. The price of refusal was the nearby concentration camps in Auschwitz, and, in the case of signing, forced incorporation into the Wehrmacht (Chromik, 2019; Król, 2018a, 2018b; Wicherkiewicz et al., 2018).

Currently, the number of Wymysiöeryś users is estimated at between 5 and 10 native speakers and 20-30 new speakers. Although this quantity may not seem impressive, these people were involved practically from scratch and their language was restored only thanks to grassroots revitalisation movements (Wicherkiewicz et al., 2018). At the end of the 20th century, scientists claimed that the first decade of the 21st century would witness the burial of Wymysiöeryś (Wicherkiewicz & Zieniukowa, 2003), which, fortunately, did not happen. In addition, a significant number of inhabitants understand the language but do not use it due to the post-war persecutions and subsequent negative attitudes toward the language, as well as to the strong influx of exclusively Polish-speaking new inhabitants of the town and the drastic reduction of traditional forms of local multilingualism. The number of people able to understand (at varying degrees) the language is estimated at around 500 people, but so far no studies have been carried out to confirm this number, apart from qualitative interviews conducted by Tymoteusz Król – one of the main activists and scientists dealing with the Wymysiöeryś language.

A small group of users and many years of qualitative research mean that the small community of Wymysiöeryś speakers, which is also focused in a small area of the town, provides excellent conditions not only for various experimental revitalisation activities but also for observing processes taking place in the environment itself. One of these is changes in linguistic attitudes and the impact of revitalisation processes on the well-being of language users and people involved in its restoration. The above-mentioned persecutions, in addition to the language, also took away an important aspect of Vilamovian identity. Identifying oneself as Vilamovian, however, is not only synonymous with the use of the language – in the case of Vilamovians, ethnicity is also expressed through a separate folk costume. Well-being indicators are also different, as, on the one hand, they are strongly influenced by historical trauma. On the other hand, the Vilamovian ethos related to self-awareness of separateness recognises that recent historical processes and the loss of local multilingualism are crucial for the current forms of community well-being. Our main goal has been to better understand the impact of language loss on well-being and its indirect impact on the loss of identity, as well as how this negative process can be reversed by linguistic and cultural revitalisation. By adopting the rules of charting, one can assume vertical (general language documentation programs and external ideas to the bottom-up, decolonising involvement of language users) and horizontal issues (the loss of language and the resulting problems with identity, well-being and trauma, on the other hand, and the impact of revitalisation and the restoration of language on the well-being of users on the other).

Although there are so few speakers, a separate identity is rooted much more widely and strongly in the community, and is based on more pillars than the language itself – regional clothing, a sense of separate origin, the vitality of tradition, and emic knowledge are crucial to maintaining a separate identity; this, when cultivated, supports the maintenance of the language.

## State of the art

An essential background of the research reported in this paper is the grassroots revitalisation movement linked to the Wymysiöeryś language, involving numerous young people in the community, and the effects of their work so far (Król, 2016b; Olko et al., 2016; Wicherkiewicz et al., 2018). The latter shows that the social impact of such community-driven and often spontaneous activities may have a greater impact than planned, formal revitalisation efforts (see Szlachta-Ignatowicz & Wicherkiewicz, 2019, p. 100).

Due to its mimetic qualities, theatre is a common language acquisition (and thus also revitalisation) medium (Winston & Stinston 2016). The scenic use of language may be more naturalistic than in pre-prompted discussions in class on a given theme. Scenic movement is a form of total physical response facilitating the connection between words and their meaning. In cultural revitalisation, theatre also entails the possibility of telling traditional stories in an engaging way and processing challenging situations which are part and parcel of being part of a minoritised community – in this way, theatre becomes a kind of art therapy.

Language revitalisation movements have long made use of drama techniques. To name only a few examples: the Hawaiian language revitalisation movement produced its first play in 1995 and went on to create a vibrant theatrical community with multiple performances centred on traditional topics. A chapter by Baker (2018) discusses this in detail, contrasting such an active approach with the static one represented by pictorial books. Driskill (2003) tells the story of revitalising Cherokee via a form of socially engaged theatre by minorities subjected to oppression and trauma, which engages participants in interactive activities aimed at understanding and counteracting social oppression. In addition, this emphasises how effective it is to engage the community in the process of restoring language through creative and interactive forms of self-expression, i.e. as a form of therapy. Another vibrant example is Hul’q’umi’num’, whose theatre project Hul’q’umi’num’ Heroes: Reclaiming Language through Theatre, with its emphasis on tradition and traditional cultural forms, was described by Sadeghi-Yekta (2020). Their situation further resembles the Vilamovian case in that the COVID-19 pandemic has caused them to stop their theatrical programme for the time being. This is why their ‘Indigenous Theatre Festival Reawakening Language on Stage’ could take place only in September 2022. The press release on this festival (Threlfall, 2022) includes the following quote: ‘We’re fighting for our language – we don’t accept it to be extinct’, which fits in precisely with the spirit in which the Vilamovian plays are being staged.

The state of research on various topics related to Wilamowice has been comprehensively documented in two annotated bibliographies (Król et al., 2020 for the years 2001–2020 and Król et al., 2022 for the years 1945–2000 and 2020–2022). Therefore, we shall only mention the texts which deal directly with the plays staged by the *Ufa fisa* troupe. The first one is an edited transcription of a discussion among activists dealing with minoritised languages in the current territory of the Republic of Poland, held on 1st December 2018: ‘With smaller spoon you enjoy it longer. A discussion on language and literature’ (Mętrak, 2019). This debate included some perfunctory mentions of literature as a basis for theatrical activities – including the first two plays in Wymysiöeryś, *The Little Prince* and *The Hobbit*. The next two texts are from 2019 and were both (co-)written by Tomasz Wicherkiewicz, who had (among other works) previously edited and published the most famous piece of literature in Wymysiöeryś – Biesik’s *Uf jer wełt*. The first of those texts (Wicherkiewicz, 2019) describes the entirety of Wymysiöeryś writing up to that point, including a summary of the *Ufa fisa* performances in order (i.e. the aforementioned *The Little Prince* and *The Hobbit*, as well as *Uf jer wełt*) and a short analysis of *Uf jer wełt*, mainly in the context of the author of the poem and the work itself in the original, taking the topic of ethnolinguistic vitality into account. This article puts the plays soundly in the context of the Vilamovian literary tradition. The second article, co-written with Justyna Szlachta-Ignatowicz (Szlachta-Ignatowicz & Wicherkiewicz, 2019), is a very precise critical analysis (termed a ‘review’ in the text itself) of the then newest play *Ymertihła*. The text finds itself located in the cadre of both Vilamovian literature and Boal’s Theatre of the Oppressed.

The year 2019 also marked the time when the most famous revitaliser of Wymysiöeryś, activist and academic Tymoteusz Król, co-wrote with Robert Borges a paper on the revitalisation of Wymysiöeryś, terming it a ‘reviTEATRalisation’ (Borges & Król, 2019). This text mentions the engaged paradigm of the activities working towards the vitality of the language through the prism of *Ufa fisa.* It talks about the beginnings of the troupe and the general use of Wymysiöeryś in public after the war, later focusing on *Ymertihła*, with a description of the plot, and *Ojeruma* – with just a short overview. In the case of the latter, it highlights the opinion-forming role of the play and how it was an expression of young people’s stance against homophobia. It focuses on the young actors as new speakers of the language – showing in detail how the theatrical group supports the learning and transmission of Wymysiöeryś. The 2021 edited book entitled *Revitalizing Endangered Languages: A Practical Guide* included a very short ‘capsule’ entitled ‘Art, Music and Cultural Activities in the Revitalisation of Wymysiöeryś’ by one of the authors of the present text (Majerska-Sznajder, 2021), which presented the activities of the *Ufa fisa* troupe as a practical example accompanying the chapter on ‘Art, Music and Cultural Activities’ by Genner Llanes Ortiz (2021).

The last text to mention on the theatre in Wymysiöeryś was also published in 2021 (Małanicz-Przybylska, 2021); rather than being based on independent research on the topic of Vilamovian theatre, it contains the author’s reflections on the topic of ‘intensive work of heritage’, gathered while she was working on the documentation of Vilamovian music. The text is relevant here because it starts by describing *Ymertihła* and then circles back to a short story of the troupe presented without proper knowledge of the context. This text has a profound problem with lack of anonymisation. The language activists are all but outwardly accused of being crafty people who came up with an effective strategy of building their own brand based on historical trauma. The fact that *Ymertihła* is based on local histories and memories is questioned and belittled – to the point where the author insinuates that since she only heard a comparable story from one elderly lady, the whole play must be a consciously created ‘own vision of the world’ (Małanicz-Przybylska, 2021, p. 94).

The present text, based on the close synergy of emic and etic perspectives and positionalities, is a first attempt to systematically examine the multi-faceted significance of the revitalisation theatre in Wilamowice as well as its role in identity and community (re)building. We also discuss the qualitative and quantitative feedback of community members with regard to the theatrical performances and other related events aimed at the reclamation of Wymysiöeryś.

### Data and methods

The methodology applied in the analysis of the theatrical plays stems from a philological approach, blending historical literary criticism with a close reading of the relevant fragments of the works. The performances are set primarily against a backdrop of contemporary developments in the revitalisation of Vilamovian culture, but the description of the circumstances of their creation is broadened by including significant parallel circumstances at a more global scale (such as the adaptation history of *The Hobbit* or the proliferation of translations of The Little Prince). The additional analyses focusing on the reception of the plays in the community and their local impact are based on emic participant observation as well as qualitative and quantitative research on the impact of new speakers (whose sense of identity has been significantly strengthened) and their artistic activities on the sense of identity of Vilamovians. This paper relies on the examination of data generated within two research projects. The qualitative sections are based upon semi-structured interviews collected in Polish and Wymysiöeryś within the *MULTILING-HIST* project (funded by the European Research Council, grant agreement No. 101002696) and the quantitative analysis on data gathered in the *Language as a cure: Linguistic vitality as a tool for psychological well-being, health and economic sustainability* (*LCure*) project (funded by the Foundation for Polish Science, grant agreement TEAM LCure 2016-3/28). The ethical clearance of the *MULTILING-HIST* project was issued by the Ethics Commission of the Faculty of ‘Artes Liberales’ University of Warsaw and by the Association ‘Wilamowianie’ in 2021. Informed consent of the respondents was assured via comprehensive oral and explanations tailored to the research participant. In the case of any audio capture of interviews, explicit consent of participants was also recorded at the beginning of each recording. The ethical standards of the *LCure* project were approved by the Ethics Commission of the Faculty of ‘Artes Liberales’ University of Warsaw in 2018. Informed consent of the respondents was assured via the inclusion of an easily understandable and GDPR-compliant clause at the beginning of the survey. In total 191 local participants completed the quantitative survey performed in the end of 2018 and the first part of 2019. Community-based experts were invited to participate in the design of the questionnaire, and much of their feedback was taken into account. Unfortunately, the social psychologists who participated in the project insisted on the mechanistic incorporation of some unmodified ‘established’ and ‘widely validated’ tools (psychological scales), employed earlier in different Western contexts, and after initially voicing our concerns, we included some of those tools. On the one hand, community members had difficulty understanding many of them, and on the other hand, the ecological adequacy of some for this cultural, historical and social context was limited. As a result, we decided not to rely on the results associated with these ill-fitted tools in any research analyses or, in cases where it was absolutely necessary, we subjected them to rigorous criticism before using them. However, the specific tools reported in this paper were created by community-based experts, including the main author of this paper, and they proved to be highly reliable. Moreover, given the community feedback regarding the challenges associated with the quantitative survey, we decided to limit the originally planned second stage of data collection that formed part of the longitudinal research design, and abandon quantitative surveys of this kind in our future research activities with the community. Rather, we focused on continuing in-depth emic qualitative interviews, carried out by the main author of this paper and a careful triangulation of different datasets, recognizing the limitations and possible biases of the quantitative data. This self-reflective, mixed-method methodology provides the base for the research reported in this paper.

## The theatre troupe

### Before the beginning

Immediately after the ban on the language in 1945, local social activist Jadwiga Bilczewska-Stanecka founded a regional folk dance and song ensemble that maintained the culture of Wilamowice under the pretext of giving stage performances. To make this possible, the narrative presented to the authorities assumed that the Vilamovian culture was ‘a mixture of Flemish-Scottish-Ukrainian-Turkish thoroughly melted in the crucible of Polishness’ (Wilamowski pierzowiec 1969). Vilamovian identity has thus been very politically instrumentalised and underwent folklorisation, understood as a shallowing and diminishing of its importance. The inhabitants of Wilamowice succumbed to such pressures both from outside and inside. The ensemble might have been presented as a place where the Vilamovian identity was maintained on stage, but from the local perspective this was mostly an optimistic façade. The local dress could only be used on stage in later years -1950s – and it was completely forbidden to present the language alongside it. Still, even this helped in the survival of the culture and thus the ensemble became the only place where community members could have intergenerational contact and maintain elements important to their identity.

To this day, the activities of this ensemble that went beyond stage performances (as its members embrace the Vilamovian culture as a part of their daily life) are perceived by other, uninvolved community members as an indicator of local well-being, and the continued existence of the ensemble gives the community a guarantee that the culture will be preserved for the next generation. It was on the basis of this group that a group of young revitalisers arose, who, out of a desire to deepen the group’s activities, began working on the documentation and popularisation of elements of Vilamovian culture. Their actions resulted in a feedback loop: the more the Vilamovian identity was presented to outsiders through the regional ensemble, the more precious and expressive it became for community members who began to get to know it again after the ban.

The development of Vilamovian theatre is inseparable from the history of revitalisation activities. Not only do the repetitions and performances themselves serve an important practical role in providing a space where the younger generations can practise the language, but every piece has premiered during a language-related event. The audience thus gathered was therefore primed in diverse ways for various aspects of each play. This is why, following a chronological order, we will describe the circumstances of the creation of each piece and the staging of its premiere, along with the results it brought about – both within the Vilamovian community and among the broader public. The latter are invariably painted with cruder strokes but this doesn’t diminish their importance to the general perception of Vilamovians as a distinct ethnic group and of Vilamovian as a fully fledged language in its own right.

### The Little Prince the first (2014) – staking a claim

The decision to stage a play in Wymysiöeryś was motivated by activists wanting to decolonise the Vilamovian identity – and doing this by venturing beyond the framework of the regional dance ensemble and the selective, instrumentalising approach associated with communist times. It was important for them to show that the Wymysiöeryś is not only a medium for historic songs and dances (for which it has been famous in recent decades), but that it is a full-fledged medium of communication suitable for telling stories about things unrelated to local folklore.

The opportunity for this presented itself via the international conference ‘Endangered languages. Comprehensive models for research and revitalization’ which we organised in 2014, aimed not only at a meeting of experts on language revitalisation, but also at strengthening the community and changing the local language policy towards Wymysiöeryś. Young activists, consisting of school students at that time, decided to adapt Antoine de Saint-Exupéry’s *The Little Prince* as a play. The choice of the story was not without prior consideration and persuasive motivation. This short children’s book, with very recognisable watercolour pictures by the author and relatively simple phrasing, was a well-known tool used worldwide to belie ideas about language homogeneity (Mętrak et al., 2025 includes a detailed analysis of this book’s role in the linguistic communities of three collateral languages of Poland, i.e. Kashubian, Silesian and Masurian). Numerous translations have been published, in a huge part due to the activity of Verlag Edition Tintenfaß. ‘Having’ a *Little Prince* in existence in the language was a useful aid in claiming Wymysiöeryś’name as a separate language in the eyes of the broader public. However it should be noted that in Poland at that time, the only language other than Polish to have a translation was Lemko – with *Малий Прінц* having been published just a year before, i.e. in Saint-Exupéry, 2013.

The formulaic nature of the story, with the Prince’s stop-overs on a sequence of planets, also allowed for a simplification of the staging and actor movements, thus enabling a low-budget adaptation that could be staged in one of the bigger rooms of the local restaurant. The premiere took place on 12th June 2014. Although the young actors from the newly created troupe had problems with remembering the text and the staging was amateurish – in the vein of a school performance – the participants delivered an important message: that Wymysiöeryś still exists, is growing stronger, and that the Vilamovian identity is being inherited by the next generation. However, the performance was not a complete separation from the past and tradition. It was the beginning of the creative use of folklore elements by young people, for instance through the colours of the Austro-Hungarian flag in the decorations, the use of nicknames, and even the creative use of traditional clothing as elements of character costumes.

To the public at large, the existence of a Wymysiöeryś language version of *The Little Prince* (even if only in the ephemeral version of a theatre play – with the only long-lasting record being a video uploaded to YouTube) became the most effective way for this community to stake their claim to the title of a language, in the absence of a Weinreichian army and navy. The Vilamovians having ‘their own *Little Prince*’ was rhetorically equivalent to having ‘a separate language of their own’ – and that was a public relations foundation that further plays could build on.

### The Hobbit (2015) – gathering a party before venturing forth

The informal group went on to prepare another play, this time for an event called ‘#Wilamowice mówią’ (‘Wilamowice speaks’), an event devoted to the promotion of linguistic visibility, developed as part of the project ‘Creation of a Tourism Cluster in the Municipality of Wilamowice on the Basis of Wymysiöeryś’ on 21st November 2015. It was then that the troupe took on the Vilamovian words *Ufa fisa* as its name. This phrase, meaning literally ‘On [its] feet’, originated from a linguistic joke among the group’s members, who also belonged to the regional dance ensemble and who paraphrased part of the lyrics of one of the Christmas carols; the members took to it mostly because it fit in with the ideology of strengthening the language. The now formalised group carefully considered the topic of their next performance. It needed to be one which would not require any sort of translation to be accessible to the public. This meant that the content would have to be known to everyone. The choice of J. R. R. Tolkien’s *The Hobbit, or There and Back Again* was based upon this particular premise as, at the time of the creation of this Vilamovian play, the last of the three films by Peter Jackson was just past its cinematic run (its Polish premiere took place at Christmas 2014 and it stayed in cinemas through the first part of the year 2015). The original author’s philologist stance – i.e. as a lover of languages – was also a point of consideration, as the Vilamovian activists were of the opinion that, were he alive, J. R. R. Tolkien would have enjoyed learning about a tiny language from the Germanic family which was so dear to him.

Conversely, the external popularity angle was attractive to some young Vilamovians, who might not have become involved had the play been something less well-known than *The Hobbit*. Such a pleasant first-hand engagement with a pop cultural adaptation led to them acquiring some Wymysorys – even if they first started using the language only in a symbolic form, as actors repeating rote texts. At the same time, this served to highlight the role of the members of the oldest generation, who were to become the main interpreters of what was happening on stage. In this way, the Vilamovian *Hobbit* showed the youngest generation that Wymysiöeryś can be used for modern content and the eldest generation that the language can be used outwardly on stage in a very publicised way (as *The Hobbit* was a much bigger publicity deal than *The Little Prince*).

The movie played a very important part in the adaptation – the costumes were inspired by it (with some Vilamovian twists), eschewing Tolkien’s colourful dwarf hoods as their defining characteristic. Moreover, the Jacksonian insistence on foregrounding the story of dwarves travelling to Erebor to reclaim their homeland from the dragon resonated perfectly with young people from a community where land ownership was rendered precarious by the vagaries of malicious 20th-century regimes. Knowing people who had been expelled from their homes by their neighbours made it easy to empathise with a similar situation – even if the one doing the expelling in the fantasy was an avaricious dragon and not an (equally avaricious) neighbour. Howard Shore’s musical score also served as the soundtrack to the play – including Vilamovian renditions of the *Misty Mountains* (from *An Unexpected Journey*) and *I See Fire* (the closing credits song of *The Desolation of Smaug* – originally written and performed by the English singer-songwriter Ed Sheeran, and quite popular in Poland).

However, the latter, entitled *Yh zoh fojer*, was not an exact translation of the original. The youth, like the lyrical subject who, in the original laments, the loss of the dwarf home of Erebor, centred the narrative on a lamentation for their culture. The text includes references to displacement and language loss:

**Table:** 

Zit uf łiht ym fonster	Didn’t you see the light in window
Fur ferłona hyt?	Of the forbidden house?
Ynzer śpröh śun śtjyrwt	Our language is dying
Zej gejt y fynsternis	It has gone into the darkness.

The song expresses hopes for a better future for their culture, which now found itself in a difficult situation:

**Table:** 

Dos ej kȧ end fu ynzer kistiöeryj	It is not the end of our story
Zejn uf di noja wymysioeryśa fłȧk	Just look at the new Wymysioeryś outfit
Nojy höfnan łiöeht wi ȧ śtam	New hope shines like a star

The last stanza directly encourages people to join language activism and shows that young people are creating a new identity for themselves.

**Table:** 

ȧn yta kum myt yns,	And now come with us
Yh wa ȧjłata dejh	I invite you
Uf dos łiwy łand, wo mȧj	to our beloved land, which
Bridyn hon łiw	my brothers have loved
Wen der hymuł cyrykziöen	When the heavens will answer
Dy gyrȧhtikȧjt wyt nȧj gejn	and justice will appear,
Ołdy cȧjtȧ wan ny kuma	The time that passes won’t return
Oder höfnan wyt błajn śtejn	but the hope will stay with us

Thanks to the popular melody and catchy lyrics, as well as the fact that it is the first cover of a pop culture song in Wymysiöeryś, which had previously only been used for historical and traditional songs, the song quickly went beyond the troupe. From then on, each performance of the *Ufa fisa* troupe featured a song. Each one was musically a cover of an existing tune but conceptually an original piece, with the lyrics reworked to the Vilamovian case. This functioned as a form of art therapy – going beyond the theatrical framework and providing the opportunity to express both frustrations and a sense of community with other members. Thanks to this, the young people acquired a sense of agency – the usage of universally known pop songs removed the veil of faux mysticism laid on Wymysiöeryś, which was an element of colonial on-stage folklorisation. This was the first step the youth took towards processing their community’s historical trauma via a theatrical performance, and also led to the first Vilamovian performers of modern pop music forming the *Biöetuł Band.* Their performances would later form an important part of various language revitalisation events alongside the troupe’s plays.

*Ufa fisa* also included Easter eggs from the local reality in the content of the plot – quotes from characters and behaviour corresponded to actual people from the community. The act of sharing a community of knowledge made it obvious to the inhabitants of Wilamowice what situations were being alluded to. The artistic angle in itself was also of importance for the young creators. They prepared a large scale mechatronic model of the dragon Smaug which went on to be praised in various reviews.

The staging of *The Little Prince* had already shown the public at large that Wymysorys is a language. *The Hobbit* further reinforced it. The language could be used to tell the story which every pop culture-conscious Pole had encountered in the previous months and years. In this way the advertisement campaigns for the Peter Jackson film series were co-opted to serve the good of the Vilamovian community in the public eye. However, the Vilamovian *Hobbit* was also relatively widely advertised before the performance – including in the regional newspaper *Dziennik Zachodni (*2015) and on the most important Polish news portal on Tolkien, *Elendilion* (Lord Ya, 2015). The latter went on to produce a full-length journalistic account of the performance (Derdziński, 2015) – which in all likelihood contributed to the fact that the performance in Wymysorys won second place among ‘Tolkien Events of the Year 2015’ in a vote held among fans in Poland (Lord Ya, 2016). In the following year the production had a one time guest performance at the Polish Theatre in Warsaw (the first of a total of three so far) on 26th February – both as a way of changing the public view of Wymysiöeryś in the country’s capital and as a celebration of International Mother Language Day. In the years that followed, this annual observance aimed at strengthening the visibility and vitality of the language; changing language policy went on to become a yearly event in Vilamovian language revitalisation activities, with a different play being performed each year.

### Uf jer wełt (2016) – exploring an afterlife

In the early autumn of 2016 a two week field school was held in Wilamowice which gathered academics and activists dealing with endangered and minority languages from around the globe. On 25^th^ September they were all treated to the very first play stemming in its entirety from the Vilamovian culture, rather than being an adaptation of a work external to Wilamowice: *Uf jer wełt* (In another world). This poem by Florian Biesik (wym. *Fliöra-Fliöra*) is the best known and longest work of Vilamovian literature and tells a story similar to that of Dante’s *Divine Comedy* – only instead of 14th-century Italians, it is the early 20th-century Vilamovians who find themselves assigned to Hell, Purgatory and Heaven. It was chosen by the troupe as a reaction to an idea by some of its members of adapting Aleksander Fredro’s *The Vengeance* (pol. *Zemsta*). This comedy is compulsory reading during Polish classes attended by every schoolchild in the Republic of Poland. Moreover, among the few plays on the compulsory reading list, it distinguishes itself by not being esoteric and inaccessible – unlike the translations from Ancient Greek theatre or the Polish Romantic tragedies with their reams of footnotes required to understand the political climate of the early 19^th^ century. *The Vengeance* is, by contrast, a relatively easy, pleasurable and even funny read – so the suggestion of adapting it was an understandable one. Yet it was as far away from the Vilamovian historical experience as possible – a story of two Polish nobles arguing about their shared castle didn’t fit the historical context of a Germanic merchant community living in a tiny town. Staging a Polish theatre classic would also undermine the decolonising approach so dear to the ethos of the troupe.

So another classic was chosen – a Vilamovian one, very recognisable due in no small part to the doctoral thesis (and its later book version) of Tomasz Wicherkiewicz, who produced the *editio princeps* of *Uf jer wełt* (Wicherkiewicz, 1998, 2003). It is with him that the stage version starts – and ends. This added framing device tells the story of how the manuscript was found and sets the stage for a historical tale of Wilamowice. The middle part, all taken from Biesik’s œuvre and life story, spans the time from the late 19th to the beginning of the 20th century. A special focus is laid on the conflict of the writer with his brother, Hermann, who famously authored a book on Wymysorys and termed it, in the very title, a ‘German patois’ (‘deutsche […] Mundart’ – Mojmir et al., 1930).

Apart from the framing device, a musical interlude was added. This took the form of a dance macabre, performed in part by characters from the piece and in part by figures in black robes with pictures of simplified skeletons glued onto their clothes. The choreography was adapted from one of the ensemble’s dance routines. This had the double benefit of shortening the practical time needed for the actors to integrate it with the rest of their scenic movements, and of playing into the ideological side of the troupe’s revitalisation activities, since it was a reimagining of how Vilamovian dances could be used in a broader context. The accompanying music was sourced from the track *Double Trouble* or *Something Wicked This Way Comes* from John Williams’ soundtrack to the movie *Harry Potter and the Prisoner of Azkaban* and was played twice during the piece – once without lyrics during the dance macabre scene and a second time, with lyrics, at the end of the piece. Williams’ adaptation of the witches’ scene from Act I of Shakespeare’s *Macbeth* was replaced with an original Vilamovian text, which was a reflection on the *topoi* of *vanitas* and *memento mori*. The use of a recognisable piece of music from a well-known franchise was yet another factor in increasing the eagerness of the local youth to participate in the play.

Having learned that Wymysiöeryś is not only a language but one with the capacity to creatively engage with present day pop culture, the public at large was up for a history lesson. The rhetorical purview of *Uf jer wełt* was one of literary antiquity – and continuity. Wymysiöeryś not only had a vibrant present but could draw from a past well of literary texts. In a country where language teaching at school emphasises engagement with a compulsory list of works of literature, the existence of a separate canon conveyed a *gravitas* of its own. *Uf jer wełt* went on to be staged again during the 2017 International Mother Language Day celebration in Wilamowice and thus started a tradition wherein, each year, a play would form both a linguistically vital and temporally substantial part of the event held in the town itself.

### Ymertihła (2018) – writing a story of one’s own

Biesik’s story, with its added framing device in the form of Tomasz Wicherkiewicz’s finding of the manuscript, touched on the extremes of the 20th century – the beginning with *Uf jer wełt* and the end with the young doctoral student just starting his academic career. The intervening years, so tragically ‘interesting’ in European history (Vilamovian history included), remained untold. The members of the troupe decided to tackle this subject next – with a story of their own.

This new scenario, meant to serve as a way of working through the traumatic past, was written collectively by the *Ufa fisa* group and was based upon various stories and experiences they had encountered while communicating with the elder members of the community who had lived through those trying times. The play, long unnamed, finally came to be called *Ymertihła* – also a first in that, even when translated into other languages, it requires an explanation. The eponymous *Ymertihła* is a type of shawl, one very precious to the Vilamovians, as the plaid from which it is made is seen as part of their multiethnic heritage. As it resembles a tartan, it rarely goes without possible ties to Scotland being mentioned. This long piece of female clothing is put on top of all other layers and is thus a clearly visible outward sign of Vilamovian identity (such that nowadays, those Vilamovian women who don’t wear the whole traditional dress for some occasion or other may still decide to use the shawl as a symbol of their identity and distinctiveness – see Chromik et al., 2021). In the piece it acts as a thread connecting all the scenes from the life of the main character, a Vilamovian woman who suffers through the historical traumata of the 20th century. She makes it through the war and the German occupation, the Soviet Russian ‘liberation’ and the subsequent introduction of the communist regime in the People’s Republic Poland, to the cold indifference of polonised youth in the newly capitalist and free Poland of today. And yet she never surrenders, she stays a proud Vilamovian safeguarding her language and identity – even if she stops wearing the traditional dress and her shawl ends up as a museum piece.

Such determination in the face of adversity was of course in part a rhetorical device used by the young coauthors to underline the importance of the revitalisation process. Yet it was also steeped in the factual evidence they had of their elders holding tightly to a culture seen as a threat or a burden by those around them. Working on *Ymertihła* therefore also left a strong impression on its creators. They recount dreaming about the play – including having dream visions of the deceased members of the ensemble, often those whose lives were used as source material that was reused in the story and who continued their calm opposition vis-à-vis a world which wanted to squash Vilamovianness under its boot. The language also played a crucial role in the play – the gradual real life transition from Wymysiöeryś to Polish was reflected in the passage of scenes and in the diminishing presence of the former in the mouths of the actors. Yet special care was taken to ensure that the play would end with Vilamovian culture not only in a museum (where the final scene takes place – with the eponymous *Ymertihła*/shawl draped on the shoulders of a display mannequin) but alive, present and ready to throw hands. *Ymertihła’s* main music theme was adapted from *The Hunger Games* movie series (more precisely from its third instalment *The Hunger Games: Mockingjay – Part 1*) with a translation of *The Hanging Tree* song featuring as the final scene. This murder ballad turned protest song is – again – textually adapted to the Vilamovian context but its association with revolution and rebellion against the externally imposed cruel reality hits just as hard. Moreover, the scene is designed to be as haunting as possible – with all the stage lights darkened and flickering (LED) candles springing to life in the hands of the performers as the only source of gradually increasing illumination. The message is clear: of individual actions becoming a positive spark in the darkness and pushing others to engage and oppose the external detrimental factors.

This very first Vilamovian theatre text written from scratch for the *Ufa fisa* group marked a moment when the broader public could see that creativity of the group members extended beyond adaptations and well into their own original writing. The new public relations gain was thus the message that Wymysiöeryś is a language with literary texts being produced in the here and now. Even while continuing the tradition of staging a play for International Mother Language Day (in that case 24th February 2018), *Ymertihła* set a precedent of this day also being the date of a premiere.

### Ojeruma (2019) – a satyr play by any other name

*Ymertihła* was such a resounding success that the troupe’s members felt stifled by the high expectations raised by their previous performance. When the time came to create a new play to be performed during the International Mother Language Day celebrations on 23rd February 2019, they finally decided on a complete genre switch. The previous two plays on Vilamovian topics had been sombre and it was deemed that the time had come to let in a bit of light and joy, so as to show that there are other facets of Wilamowice than trauma. So the play would be a comedy. However, the troupe enjoyed the idea of showcasing Vilamovian history and decided to fill in a gap – the times before *Uf jer wełt*. Taking a leaf from their previous story, they used a Wymysiöeryś world *Ojeruma* as the main part of the title. This interjection is specific to Wilamowice and in essence carries a similar meaning to the English y*ikes*.

The main character was also based upon a uniquely Vilamovian concept – being *fłymiś* (literally ‘Flemish’, as a common Vilamovian view of their origins has it that they came from Flanders). The young male lead might be a bit awkward and maladroit but he is at the same time crafty, ingenious and enterprising (all qualities highly appreciated by the merchant society). Those features allow him to grow his good fortune – a faculty stereotypically required of a Vilamovian man in want of a wife. Stereotypes like this one were the very building blocks of *Ojeruma*. Much like *Ymertihła* was built upon stories that the young members of the troupe had heard from elder Vilamovians, *Ojeruma* was constructed from clichés about the community. The play worked through such topics as the matriarchical power structure of the group, and Vilamovians being (in)famously connected with the cabbage trade (both in jest and as a form of soft persecution and ridicule). The canvas on which they were painted was one of mercantile activity – travelling around Europe buying objects for lower prices and selling them for higher ones. Once again the Vilamovian traditional dress featured as a prominent fixture of the story – with the tartan fabric being seen in the cadre of the play as being imported directly from Scotland. Among other places visited by the main character and his sidekick (his younger sister) were Istanbul (the Eastern trade periphery to Scotland’s West) and Vienna (the Austro-Hungarian Habsburg imperial centre, so close to the Vilamovians’ hearts – not least because of their diaspora, who live there to the present day).

The plotting didn’t stop at Vilamovian emic beliefs and legends but was also built so as to interweave other important threads of the local identity and smaller contemporary anecdotes. The local official buys his daughter’s suitor a car so as to be left alone and in peace, while the local youth worry about climate change and continue subtle proclamations of solidarity with LGBT folks throughout the play. All of these threads refer to contemporary happenings in Wilamowice (including the despicable anti-LGBT ruling which was in force when *Ojeruma* was just being staged; see Majerska-Sznajder, 2022). The comedic shape of *Ojeruma* and its inescapable connection to the tragically sombre *Ymertihła* resemble the connection of Athenian satyr plays with the tragedies of that age – both performed during the city Dionysia and often addressing the same subject from different angles, meant to evoke strong emotions of various kinds. The two Vilamovian plays function in a comparable way and their reception followed that reasoning. *Ojeruma* was seen as showcasing the fact that Wilamowice, its history and language can also be presented in a fun – and funny – way, that the inhabitants can reclaim the jokes made about them and turn those jokes into an empowering story about surmounting obstacles and proving themselves in the world.

### The Little Prince the second (2020) – closing the circle

The first day of 2015 marked the moment when the works of Antoine de Saint-Exupéry, and among them *The Little Prince*, entered the public domain in countries that followed the 70 year rule (in France the copyright got extended for another 30 years due to the death being designated as having been for France, i.e. *mort pour la France*). This instantly simplified the process of translation and publication, as there was no longer any need to arrange the legal part of the endeavour. A veritable flurry of activity followed and in April 2017 *The Little Prince* became the world’s most translated non-religious book – with at least 300 different language versions extant (AFP Relax News, 2017). As of now (3rd February 2024) it has been documented to have been published in ‘570 different languages and dialects’ (Sandoz, 2024) – leading to the possibility of such publications as *The Little Prince* in 29 German varieties (*Der kleine Prinz in deutschen Mundarten: Jubiläumsausgabe*, Saint-Exupéry, 2023b), 32 languages of France (*Le Petit Prince en langues de France. Métropole et outre-mer*, Saint-Exupéry, 2023c), 30 ‘French local languages’ (*Le Petit Prince polyglotte en francophonies: Édition anniversaire*, 2023d), and even in multiple different unrelated languages, one per chapter (*Le Petit Prince polyglotte: Édition anniversaire exclusive*, 2023e). Languages and language variants from the current territory of the Republic of Poland can easily be counted among the overwhelming numbers, with versions in Old Prussian (*Līkuts Princis*, Saint-Exupéry, 2015), the Greater Polish dialect (*Książę Szaranek*, Saint-Exupéry, 2016), Yiddish of Warsaw (*Da klayna prints*, 2017), Silesian in the variant of Upper Silesia (*Mały Princ*, 2018a), Kashubian (*Môłi princ*, 2018b) and Masurian (*Małi Princ*, 2018c) having been published in the years between the time described here (i.e. late 2018/early 2019) and the first Vilamovian theatrical production of *The Little Prince*. In 2018 the lack of a physical book version in Wymysiöeryś was starting to wear through and undermine the very propagandist success we discussed previously. This was clearly visible during the discussion on language and literature ‘With smaller spoon you enjoy it longer’ (Mętrak, 2019) held as the final part of the international conference ‘Small language – large issue’. The Vilamovian representative therein (and of the authors of the current text) volunteered a story of people asking her ‘How come you call yourselves a language and yet you don’t have *The Little Prince* [translated]?’ (p. 165). So a translation was soon arranged. This took the better part of the year 2019 (as the translators, Tymoteusz Król and Joanna Maryniak [one of the authors of the current paper], can attest) and the paper version of *Kliny Fjyśt* (2020a) was set to be available for distribution during the International Mother Language Day celebrations in 2020^1^.

The decision about what play would be performed that year was a simple one. It was the first time the play could promote a tangible product that spectators could take back home – and the *Ufa fisa* troupe seized that chance. The new Little Prince wore a *lajbik –* a specifically Vilamovian kind of vest – and the group members, now with several years of training under their belt, produced a fully rounded adaptation that gathered a thunderous applause. This performance of *The Little Prince* took place on 29th February 2020.

Then COVID came.

### A Christmas Carol (2024) – being a ghost story of Wymysiöeryś yet to come

The pandemic, which was already raging in various parts of the world, took off exactly two weeks (with the lockdown being instituted in Poland on 14th March 2020) after the second theatrical performance of *The Little Prince* in Wymysiöeryś. The culture and its revitalisation were not unaffected by lockdowns and other measures. This meant a very long pause in all activities held offline. Only now, 4 years later – just in time for the 2024 celebration of International Mother Language Day – is the theatre coming back, this time with an adaptation of *A Christmas Carol* by Charles Dickens, entitled *Kistiöeryj fum Hȧliköwyt*.

The Vilamovian Scrooge extends his scepticism beyond Christmas and general human decency – he is also a representative example of those who don’t see a point in speaking Wymysiöeryś. Of course, the ghosts change his linguistic ideology and he ends up speaking the language himself (and believing in Christmas – as every good end-of-story Scrooge should). This play also marks a return to the idea of presenting adaptations of songs as part of the performance. In this case the choice was obvious and derived directly from Dickens’ inclusion of ‘God Rest You Merry, Gentlemen’ in his story. This carol is quite unknown in Poland and so it can’t be taken for a translation of a Polish piece. Moreover, the Wymysiöeryś translation of ‘God Rest You Merry, Gentlemen’ was completed early enough that it could be a part of the 2023/24 Christmas season in the Vilamovian community. Thus, a translation made for the use of a theatre troupe has started putting down roots as an actual Christmas carol in its own right.

## Reception and impact in the community

### Qualitative feedback

Each of the performances prepared by the *Ufa fisa* troupe has had an impact not only on the young generation participating as actors, but an effect on the entire community. Its members react according to different patterns, most often related to age and declared identity. This division is typical of Wilamowice: the oldest generation that experienced persecution still occasionally wear Vilamovian dress and, despite their painful history, usually identify themselves as Vilamovians. The middle generation, brought up during the language ban, have only recently begun to discover and explore the Vilamovian identity, which had previously been largely unknown to them. Most of them, if they had the opportunity at all, began to learn about their native culture only in adulthood, when the persecutions had already subsided. However, they did not adopt it, considering it mostly a thing of the past; instead, they developed a Polish identity. Some of them, however, reveal a mixed or contextual identity, considering themselves Vilamovians in precisely defined situations. The youngest generation follows the pattern of the middle generation, but shows greater freedom and deeper understanding of their chosen identity. They consciously choose to be Vilamovian and try to creatively build contemporary attitudes based on the traditional local ethos. However, this generation also includes the largest number of individuals presenting an exclusively Polish identity, completely cutting themselves off from the community.

#### The oldest generation

The oldest generation, initially distrustful of the revitalisation processes (their reluctance was often caused by the fear of the return of persecution not only towards themselves, but also towards the youth who were involved in the language reclamation), welcomed the performances very positively. In particular, a breakthrough moment for them was *The Hobbit*, where the main idea was to highlight their role as language keepers, because people who did not know the plot during the performance asked the elders for translation, and senior members of the community were treated as honoured guests. It was a particularly positive feeling for them, because for the first time since before the language ban, apart from sporadic cases or performances by a regional band, they were hearing Wymysioeryś in a public context:

*I didn’t think that I would hear Wymysioeryś on stage in my lifetime.* (woman, ca. 95 years old)

The performances in their native language helped them believe that a change of language policy is indeed possible and gave them real hope that Vilamovian culture would not end with their generation, which was a heavy burden for many:

*Sometimes when I think that my daddy, my mommy… There on the other side they will ask why I didn’t teach them (children, grandchildren), and it doesn’t matter that they were displaced and it wasn’t allowed, but somehow we could try later and maybe it was our fault that we didn’t try hard enough, because when they no longer beat us for it, the children didn’t want it and I thought “What would they need it for?” And it’s easier on our hearts that our culture will not go with us up the hill (to the cemetery).* (woman, ca. 90 years old)

On top of the persecution-induced trauma, the members of the Vilamovian community self-victimise by blaming themselves for decisions which at a certain point allowed them to survive. Not using Wymysioeryś was such a choice necessary for survival but detrimental to language transmission. The Vilamovian elders (such as the 90-year-old lady quoted above) blame themselves for not making an effort to save the language – even though it would most likely end in an uptick in persecution for themselves. The language revitalisation process and its effectiveness is thus a source of relief for them.

The topics chosen by the youth for theatrical performances have also helped the oldest people to work through their trauma. This was particularly salient during the performance of *Ymertihła*, which the young people based on the histories of the members of the oldest generation, who recognised the situations from their own lives on stage during the performance:

*It was beautiful. I cried during the performance because not only did they play beautifully, but I remembered everything that my mother and grandmother told me (…). There were references there (…). I’m glad that it was the young who showed that they had courage and that it happened in Warsaw later, because it means that more people will know about our harm.* (woman, ca. 80 years old)

*It was just as they showed. They beat as they showed. They beat my dad to death… And the young people didn’t hide anything, they showed who was beating [us] and for what. And this is very good, let it go to the world, at least that way they will restore justice to them.* (woman, ca. 95 years old)

Thus, for the elderly, the role of the performances was to restore historical justice and provide some kind of compensation and reparation for their suffering.

#### The middle generation and the uninterested youth

The middle generation and the young people who are not interested (or not yet interested) in continuing the Vilamovian identity show a wide range of attitudes towards revitalisation efforts. Many see them as senseless activities because, in their opinion, maintaining their identity is impractical and pointless. They express this opinion in a more or less direct way:

*Well, they could organise a karate class instead of the Wymysioeryś one – it would be more useful. And apart from that, nothing is happening. In other communes they offer painting and make-up courses, but here there is nothing.* (woman, ca. 60 years old)

*I’m not going to act like a fool and make a fool of myself. If they want, they can go to a Maasai village to watch them and not make people ridicule themselves here.* (man, ca. 25 years old)

Some of them perceive the efforts of activists and young people as something useful to the general public as an active and interesting way of spending their free time, but they do not go beyond viewing these activities as an ordinary theatre hobby:

*It’s nice that you have something to do – now young people only spend time in front of computers and phones. At least this is how you spend your time creatively.* (woman, ca. 45 years old)

*You could finally do something in a normal language, in Polish, because it’s nice to watch you, but one can’t concentrate on what’s going on because they have to read the subtitles.* (woman, ca. 50 years old)

However, for some of the middle generation, topics discussed by the young serve as an additional source of knowledge about their own culture, which they could not learn about when they were younger:

*I thought I knew a lot about Vilamovian culture because I had been in FIL^2^ for so many years, but only when I watched (the performances) I was shocked at how much I didn’t know.* (woman, ca. 45 years old)

*I didn’t even know one third of what they showed. I thought they were making it up (Ymertihła), but then when they told me it had really happened, I started looking for information and finding out the fate of my own family. I had no idea in my life that it was like this, until my mother started telling me that her father had been sent to the Urals, but it seemed so normal to her, so obvious, because there were a lot of people [sent] there*. (woman, ca. 40 years old)

Community members who had been influenced by the activists’ activities began to reveal a mixed or contextual identity; over time, many of them also would declare themselves to be Vilamovians and show appreciation of the role played by the young for the survival of Vilamovian culture:

*It is good that someone is taking care of it and promoting our Vilamovian culture. Thanks to this, other young people can see that this is something valuable today and being a Vilamovian is not an embarrassment at all* (man, ca. 25 years old))

It is possible that the influence of *Ufa fisa* on the middle generation will increase because almost half of the actors engaged in the preparation of the most recent performance belong to this age group, including people who participated in the first Wymysioeryś course for adults.

#### The engaged youth

The perspectives of the youth engaged in the *Ufa fisa* activities are diverse. Many members of this group initially joined it to take part in what seemed to be an interesting theatre activity or because of their friends who were already involved. Many of them stayed for a long time, becoming committed to the preservation and learning of the language. Over time, they began to see this form of action as their duty to prevent the death of the language:

*Because you are convinced that this one action is one step away from disappearance. We succeeded once again and it makes me happy.* (man, ca. 25 years old)

Very often they are deeply moved by discovering the linguistic situation and learning about the history of culture and language loss, which triggers a kind of rebellion against this state of affairs. This is expressed in their actions:

*Well, we are working to show that the people of Wilamowice oppose the fact that no one recognises them and that they were forbidden to practise this culture. It’s a kind of youthful rebellion, so that no one tells us what we can and cannot do.* (woman, ca. 25 years old)

They often take the task to which they became committed very seriously, perceiving it as a debt they owe to previous generations, giving them, in a sense, a voice. This was felt especially strongly during *Ymertihła*:

*I dreamed that I entered my regular classroom and instead of my friends, it was just old people in their [traditional] dress. I didn’t know them, but they knew me. And they came up and shook my hand, saying thank you for finally telling this story. Even my great-grandmother came up to me. And I woke up* (man, ca. 25 years old).

An important aspect of their activity, as they see it, is that one way of restoring justice is by telling stories important to the previously marginalised Vilamovians, helping them to work through historical trauma:

*Even when I think about it now, I’m glad we made* Ymertihła*. I’m proud of it. It is like removing a bandaid and pulling off the scab. It hurts, but then there is relief.* (woman, ca. 20 years old)

They are also aware of their role in restoring and creatively adapting the Vilamovian identity for the new generation, and they express this not only through art, but also in direct conversations. For this reason, people from the community who deny their Vilamovian identity often accuse them of creating artificial phenomena and using them for their own benefit.

*People will prowl, they won’t like it, because it’s easier to show Wymysioeryś as folklore and not as difficult topics.* (man, ca. 35 years old)

Both the topics of the plays and their role in the community are often a heavy mental burden for the members of *Ufa fisa* and the activists cooperating with them. Not only are they sometimes overwhelmed by their sense of responsibility for the fate of the endangered culture and language entrusted to them (of which they feel they are the guardians), but they also often have to fend off external pressure and deal with anxiety related to the national policy towards ethnic minorities. This was especially clear during the production of *Ymertihła*, which coincided with the tightening of the Polish government’s policy towards minorities, and during *Ojeruma*, when they were protesting against nationwide anti-LGBT movements.

*The fact that we will be locked up for this performance is nothing, but what about those children who are not yet adults?* (man, ca. 30 years old)

*I’m afraid that we won’t finish playing it yet, and there will be police dogs standing in front of the building of the Volunteer Fire Department* [where the play was staged] (man, ca. 30 years old)

Despite these concerns, most of the activists managed to ‘decolonise’ their way of thinking, which has enabled the activities of the group to continue, while its members have deepened their activist engagement even further:

*We shouldn’t worry about what others will think about it, because it is our history and we have the right to it.* (Vilamovian man, ca. 20 years old)

### Quantitative feedback

The qualitative feedback has also been confirmed by a quantitative survey carried out in Wilamowice in 2018–2019 within our *Language as a Cure* project. Nearly 300 Vilamovian residents took part in the survey, which is a representative group of 10% of the entire community. They included representatives of various identity categories (those identifying themselves as Vilamovian, Polish-Vilamovian inhabitants, Poles from Wilamowice, and exclusively as Polish). We asked for feedback regarding recent language revitalisation and cultural activities, which included the *Ufa fisa* activities and the teaching of the language to children. 94.1% of respondents declared that they feel happy ‘that there are activities aimed at the survival of Vilamovian culture and language’; similarly, 87.7% confirmed that they feel happy ‘watching the efforts to keep the Vilamovian culture for the future generations’. As many as 90.2% agreed with the statement ‘I am happy because children are learning Wymysiöeryś and 81.1% with the statement ‘I feel pride and joy because I belong to the Vilamovian community’. This largely affirmatory feedback confirms the very positive reception and impact of recent language revitalisation activities among community members.

In the survey we also asked about the frequency of respondents’ participation in cultural events associated with the preservation of the local identity, e.g. the local folklore event ‘Wilamowskie Śmiergusty’, International Mother Language Day, events of the Association ‘Wilamowianie’, and membership of regional groups, including the ‘Wilamowice’ Regional Group and ‘Cepelia-Fil’, another band also presenting Vilamovian folklore, but based on the youth. Almost every respondent confirmed their participation in at least one activity. The majority (151, n = 156) declared participation in the ‘Wilamowskie Śmiergusty’, originally associated with Easter Monday, that has taken on the form of an annual festival (no longer connected to Easter). Mother Language Day and events organised by the Association ‘Vilamovianie’ gathered fewer participants (76, n = 156). Long-term engagement in cultural activities through regional folk groups was significant, including in the Regional Dance Group ‘Wilamowice’ (114, n = 156) and Cepelia-Fil (60, n = 156). When we looked at the correlations between different variables, including the involvement in these organisations, meaningful patterns were revealed. The more frequent thoughts about respondents’ Vilamovian identity were associated with participation in the ‘Wilamowskie Śmiergusty’ (0.224**), events organised by the Association ‘Wilamowianie’ (0.160*) and the Regional Dance Group ‘Wilamowice’ (0.295**); the affirmation that being Vilamovian was an important part of the respondents’ identity was positively correlated with their participation in Mother Language Day celebrations (0.167*), ‘Wilamowskie Śmiergusty’ (0.230**) and the Regional Dance Group ‘Wilamowice’ (0.203*). Finally, direct involvement in the activities supporting Wymysioeryś language and culture were positively and strongly correlated with events organised by the Association ‘Wilamowianie’ (0.376**), Mother Language Day (0.506**) and the Regional Dance Group ‘Wilamowice’ (0.259**). Surprisingly, linguistic-cultural activism was negatively correlated with involvement in the ‘Cepelia-Fil’ group (-0.221**); participation in this organisation, as well as in other local groups, was not significantly correlated with items referring to Vilamovian identity.

These results imply that not all locally active cultural associations contribute to the strengthening of local ethnic identity and involvement in language revitalisation and support activities, even if they have similar resources and officially promote local culture. Thus, the quantitative data confirm that the role played in this respect by the Association ‘Wilamowianie’ and the Regional Dance Group and their events (theatrical performances, Mother Language Day) is quite exceptional in terms of its social impact and of building local identity in relationship to the heritage language and culture; an important mechanism behind this seems to be the transgenerational composition and collaboration within these organisations, which facilitates language and knowledge transfers as well as strengthening trans-generational links important for local identity. These groups and their activities aimed at linguistic-cultural revitalisation also strongly influence the social environment by building and promoting a modern Vilamovian identity and sense of community, rooted in values from the past, but oriented toward creating stable spaces for its continuity into the future. This is clearly reflected in the significant correlations between this activist involvement and the strengthening of Vilamovian identity, which provides an important complementary dimension in the ongoing evaluation of language revitalisation efforts and their efficacy.

## Discussion and interpretation: concluding thoughts

Vilamovian theatrical activity has played a fundamental role in the community-driven language revitalisation process, creating and offering an environment in which a significant part of the community was able to find multiple positive features and encouragement to engage and relate. On top of being an outlet for creative expression (a concept highly attractive to young people), it is one of the most salient activities sustaining the linguistic vitality of Wymysiöeryś. Even if a new play is staged just once a year, with very few repeat performances (if any), the act of preparing it is still of great value and long-term impact.

The young people engaged in the work of the troupe – including choice of subject, playwriting, repetitions and the final performance – have benefited in many ways from the activities, which have become, for a number of them, a long-term commitment. They got to participate in the co-creation and renewal of their ethnic identity, as their actions extend beyond the troupe and are shared beyond the members of the troupe to other community members. This is why Ufa fisa can be seen as a community of practice following Wenger’s concept (Wenger, 1999). The collective identity of participants as Vilamovians is consolidated, reinforced and sometimes created via their participative connection in a collective, active and repetitive process. While the plays are sometimes performed outside Wilamowice (e.g. in Warsaw), the bulk of the preparation process as well as most of the performances take place in the town itself – thus being highly local. The functioning of the group is also part of an organic evolution without formal control, aligning very well with the concept of community of practice. This analysis also helps us explain why in our quantitative study the participation in other local groups (such as ‘Cepelia-Fil’) wasn’t positively correlated with the linguistic-cultural activism for the activities and models of operation of those groups poorly responds only to the community of practice paradigm and thus have less identity-forming power. Wenger also describes how the authenticity of participation ‘might be one of the most deeply essential requirements for teaching’ (Wenger, 1999: 277) and the Vilamovian theatre with its deep engagement with local culture and local knowledge transfer has been uniquely suited for that purpose. This also applies to the second essential dimension of community’s ethnolinguistic vitality, namely Wymysiöeryś, the language itself. Not only have the members of the troupe developed the currently most efficient strategy of learning their heritage language in as natural an environment as possible in a community with such a burden of historical persecutions and trauma, but they have also been actively able to face this trauma by talking about it and thus working through it. The emergence of such a possibility of debate (as in the case of *Ymertihła*, which touched upon the most sensitive aspects of the recent past of Wilamowice) is of great value to the Vilamovian community at large, as such topics fare better in spaces of discussion rather than in hushed conversations. This also allows the eldest generation to feel that their story is being heard and acknowledged, that historical justice is being restored and that the very act of voicing it and talking it through provides a kind of compensation. Members of the ‘uninterested’ middle generation may claim that the theatrical activities of *Ufa fisa* are pointless but nevertheless some of them admit to learning their own history from watching the performances.

Another lesson imparted by this theatrical activity was one of defolklorisation. Vilamovian culture, so long relegated only to the domain of colourful folkloric performances by the ensemble, found itself broadening its outreach to diverse aspects of human experience – and that was new and surprising both for the more indifferent inhabitants of Wilamowice and for outsiders. The latter group also benefited a lot from the judicious choice of topics by the *Ufa fisa* troupe. The Vilamovians, who were largely unknown outside of a very narrow circle of specialists, started to be seen as a community which has its own language with its own distinct literary history and an ability both to reflect on it critically and work through trauma with it as the medium, and to use it as an outlet for laughter. Wymysiöeryś could be used to creatively engage with modern pop culture (with the Vilamovian *Hobbit* and *Little Prince* gathering quite a notoriety online) and to sing covers/adaptations of widely-known songs.

The *Ufa fisa* troupe and its performative art have been a vital instrument in the co-creation of a modern Vilamovian identity, in language reclamation, in challenging the oppressive nation-state ideologies so much ingrained in the Global North and in highlighting the epistemological autonomy of the community. Thus, it has also contributed greatly – and continues to contribute – to the process of (re)building of the community, one so hurt by the politics of the 20^th^ century and yet so resilient as to continue to survive and grow ever stronger in the 21^st^ century.

## Data Availability

The data from the LCure project are available from the corresponding author, JMSz, upon reasonable request.

## Notes

1 For the sake of completeness, it should be noted that 5 more local Little Princes have since joined the printed throng: *Mały Królewic* (in the language of the Podhale region, i.e. Saint-Exupéry 2020b), Małý Prync (in Podlachian, i.e. Saint-Exupéry 2021 – this one only available in a digital format), *Mały Kśę¨ć po kurpśosku* (in the language of the Kurpie region, i.e. Saint-Exupéry 2022a), *Môłi Ksążã* (a second Kashubian version, i.e. Saint-Exupéry 2022b), and *Mały Ksionże po naszymu* (in Silesian, in the variant of Cieszyn, i.e. Saint-Exupéry 2023a).

2 Another folk dance ensemble presenting Vilamovian culture through onstage folklore.
